# In Vivo Assessment of Resistant Starch Degradation by the Caecal Microbiota of Mice Using RNA-Based Stable Isotope Probing—A Proof-of-Principle Study

**DOI:** 10.3390/nu10020179

**Published:** 2018-02-06

**Authors:** Elena Herrmann, Wayne Young, Verena Reichert-Grimm, Severin Weis, Christian U. Riedel, Douglas Rosendale, Halina Stoklosinski, Martin Hunt, Markus Egert

**Affiliations:** 1 Institute of Precision Medicine, Faculty of Medical & Life Sciences, Furtwangen University, 78054 Villingen-Schwenningen, Germany; elena.herrmann@hs-furtwangen.de (E.H.); severin.weis@hs-furtwangen.de (S.W.); 2 Institute of Microbiology and Biotechnology, University of Ulm, 89069 Ulm, Germany; verena.grimm@gmx.de (V.R.-G.); christian.riedel@uni-ulm.de (C.U.R.); 3 AgResearch Limited, Food Nutrition and Health Team, Grasslands Research Centre, Palmerston North 4474, New Zealand; wayne.young@agresearch.co.nz; 4 Riddet Institute, Massey University, Palmerston North 4474, New Zealand; 5 High-Value Nutrition, National Science Challenge, University of Auckland, Auckland 1142, New Zealand; 6 The New Zealand Institute for Plant & Food Research Limited, Palmerston North 4474, New Zealand; douglas.rosendale@plantandfood.co.nz (D.R.); halina.stoklosinski@plantandfood.co.nz (H.S.); martin.hunt@plantandfood.co.nz (M.H.)

**Keywords:** resistant starch, gut microbiota, RNA-SIP, *Clostridiales*, *Dorea*

## Abstract

Resistant starch (RS) is the digestion resistant fraction of complex polysaccharide starch. By reaching the large bowel, RS can function as a prebiotic carbohydrate, i.e., it can shape the structure and activity of bowel bacterial communities towards a profile that confers health benefits. However, knowledge about the fate of RS in complex intestinal communities and the microbial members involved in its degradation is limited. In this study, 16S ribosomal RNA (rRNA)-based stable isotope probing (RNA-SIP) was used to identify mouse bowel bacteria involved in the assimilation of RS or its derivatives directly in their natural gut habitat. Stable-isotope [U^13^C]-labeled native potato starch was administrated to mice, and caecal contents were collected before 0 h and 2 h and 4 h after administration. ‘Heavy’, isotope-labeled [^13^C]RNA species, presumably derived from bacteria that have metabolized the labeled starch, were separated from ‘light’, unlabeled [^12^C]RNA species by fractionation of isolated total RNA in isopycnic-density gradients. Inspection of different density gradients showed a continuous increase in ‘heavy’ 16S rRNA in caecal samples over the course of the experiment. Sequencing analyses of unlabeled and labeled 16S amplicons particularly suggested a group of unclassified *Clostridiales*, *Dorea*, and a few other taxa (*Bacteroides*, *Turicibacter*) to be most actively involved in starch assimilation in vivo. In addition, metabolic product analyses revealed that the predominant ^13^C-labeled short chain fatty acid (SCFA) in caecal contents produced from the [U^13^C] starch was butyrate. For the first time, this study provides insights into the metabolic transformation of RS by intestinal bacterial communities directly within a gut ecosystem, which will finally help to better understand its prebiotic potential and possible applications in human health.

## 1. Introduction

The large bowel of humans and other mammals harbors a complex and highly diverse microbiota [1]. Although a unique community structure is observed in each individual [2], the functional capacities encoded by this microbiota are largely shared between individuals [3]. Because of the mutual metabolic activities of host and microbiota, the gut microbiota can be regarded as a separate ‘organ’ [4] that enables the host to gain energy from substrates that are otherwise inaccessible [5,6]. Resistant starch (RS), as found in raw potatoes, is ‘resistant’ to digestion by pancreatic enzymes in the small bowel of the host and, hence, able to reach the large bowel, where it is utilized by distinct members of the colonic microbiota [7,8]. While starch from raw potatoes is considered resistant to digestion due to a high amylose content (Type 2 RS), other types of starch are resistant due to physical inaccessibility (Type 1, e.g., in seeds and grains), retrogradation caused by cooking (Type 3) or deliberate chemical modification (Type 4) [9,10]. Consumption of such non-digestible carbohydrates may selectively stimulate and enrich for bacteria that transform the fermentable substrates into diverse short chain fatty acids (SCFA) which in turn are known to positively influence host metabolism, physiology, nutrition and immune function [11,12]. As compositional changes in the large bowel community are linked to several gastrointestinal disorders, such as inflammatory bowel disease, but also to obesity, allergies and diabetes [13], stimulation of beneficial members of the colonic community to boost health-promoting intestinal fermentation might be of considerable importance for human health.

A large number of studies have shown a selective modulation of the large bowel microbiota structure by diet-based interventions [14]. However, detailed investigations of which bacterial groups are directly using RS fractions and tracking the fate of RS carbon atoms in complex intestinal ecosystems are still scarce, even though there is increasing evidence for the health benefits conferred by RS [9,15], including its potential clinical application in the therapy and maintenance of gastrointestinal health [16]. It is therefore important to define which kind of bacteria are actually responsible for starch degradation under in situ conditions, as this may lead to better predictions of community structures when being altered by dietary modifications. Previous in vivo studies have reported increases in bacterial taxa such as *Bifidobacterium*, *Ruminococcus*, *Lactobacillus* and *Roseburia* due to ingestion of RS [17,18,19,20]. However, approaches to characterize intestinal community profiles upon dietary modifications are usually biased by enrichments and are often solely based on 16S ribosomal RNA (rRNA) gene sequencing techniques that do not provide a direct link between specific bacterial groups and their ecological function in situ. Knowledge that sheds light on the dynamics of prebiotic carbohydrate utilization and its fate directly within intestinal environments is essential in order to better understand the role of the involved microorganisms and the effects on human physiology. Therefore, the use of molecular techniques that couple the phylogenetic identity of microorganisms to their ecological function are fundamental for in-depth analyses of the bowel microbiota.

The introduction of stable isotope probing (SIP) in microbial ecology represents a significant advantage in the identification of bacteria with specific metabolic capacities under in situ conditions [21,22]. This approach depends on the substrate-dependent incorporation of a stable isotope, predominantly ^13^C, into biomarkers (e.g., nucleic acids) of microorganisms that are involved in the assimilation process of interest [23,24,25]. In particular RNA-based SIP experiments [26] have gained attention. RNA shows a greater sensitivity in response to metabolic conditions compared to DNA, due to a faster synthesis rate, which is independent of cellular replication processes. This leads to a quicker labeling of RNA within shorter incubation times compared to DNA [23,27]. Initially applied to study phenol degradation processes in an industrial bioreactor [27], RNA-SIP has recently been shown to be suited to link structure and function in intestinal habitats as well and was, therefore, used for the analysis of assimilating processes of simple and more complex sugars, including prebiotics such as RS and inulin [28,29,30,31,32,33].

The aim of this study was to identify bacteria that assimilate [U^13^C]-labeled potato starch, a source of RS, in vivo in a complex caecal community resident in mice. We observed incorporation of the ^13^C-label into bacterial 16S rRNA only 2 h after oral administration of [U^13^C] starch to the mice. Phylogenetic analysis of unlabeled and labeled 16S rRNA pools revealed slight differences in the caecal microbiota structure, indicating microbial taxa involved in starch degradation. In addition, metabolite screening by gas chromatography-mass spectrometry (GC-MS) analysis showed label incorporation mainly into butyrate. To the best of our knowledge, our results represent the second report worldwide using RNA-SIP under the in vivo conditions of a vertebrate intestinal system [30], and the first one using RS.

## 2. Materials and Methods

### 2.1. Animal Information

Animal experiments were approved by the ethical committee for animal experimentation of the University of Ulm and the responsible legal authority (Regierungspräsidium Tübingen, Tübingen, Baden-Württemberg, Germany, animal license TVA1238). In total, nine 8-week-old C57BL/6J mice of both sexes were used. The animals were raised at the animal facility of Ulm University in a specific pathogen-free (SPF) environment at 21 °C and 50–55% humidity on a 14 h/10 h light/dark cycle, and provided a standard laboratory diet (61% carbohydrates, 27% protein, 12% fat; Mouse-Breeding fortified, ssniff, Soest, Germany) and water ad libitum.

### 2.2. Feeding Experiment with Isotope-Labeled Raw Potato Starch

Mice were randomly assigned into three groups of three animals each. At the start, animals of two groups received 0.5 mL of a phosphate-buffered saline solution (pH 7.0; PBS; Thermo Fisher Scientific, Waltham, MA, USA) suspended with ~0.4 g of uniformly (98%) isotope-labeled [^13^C] starch extracted from raw potatoes (IsoLife, Wageningen, The Netherlands) via oral gavage into the stomach. The placebo control group received 0.5 mL of a PBS solution (pH 7.0) without starch. Animals were sacrificed at 0 h (placebo control group) or 2 h and 4 h after starch administration by cervical dislocation and the entire gastrointestinal tract (GIT) was immediately dissected post mortem using sterile surgical instruments. The caecum section was opened individually, and contents were collected from each animal. Samples were resuspended in 1 mL of sterile RNAlater (Qiagen, Hilden, Germany) and stored at −80 °C until further analyses.

### 2.3. RNA Extraction, Isopycnic Ultracentrifugation, Gradient Fractionation, Reverse Transcription

Bacterial cells from all samples were pelleted for RNA extraction by centrifugation for 20 min at 4 °C and 3220× *g*. To remove residual RNAlater, pellets were washed with PBS and centrifuged again. Total RNA from ~0.1 g of caecal material was extracted and purified from co-extracted genomic DNA as previously described [32]. RNA integrity was checked with an RNA 6000 NanoLabChip using an Agilent 2100 Bioanalyzer (Agilent Technologies, Inc., Santa Clara, CA, USA) and quantified with a Nanodrop ND-1000 spectrophotometer (Thermo Fisher Scientific, Waltham, MA, USA). Absence of DNA was verified by PCR (Mastercycler Pro S, Eppendorf, Hamburg, Germany) targeting 16S rRNA genes using the universal bacterial primer pair F_Bact 1369 and R_Prok 1492 [34].

To determine the general caecal microbiota structure in each individual mouse prior to the density dependent separation via isopycnic density gradient ultracentrifugation, total caecal RNA from each mouse was converted to complementary DNA (cDNA) in technical duplicates by using the SuperScript VILO cDNA Synthesis Kit (Life Technologies, Camarillo, CA, USA) according to the manufacturer’s instructions.

In order to identify bacteria distinctly involved in starch degradation, RNA from individual mice was pooled according to experimental group (control, 2 h, and 4 h of incubation with starch) in equal ratios to yield ~600 ng per pool, respectively. Subsequently, each RNA pool was separated by isopycnic density gradient ultracentrifugation according to their buoyant density as described elsewhere [31] with minor modifications. In brief, RNA was added to a cesium trifluoroacetate (CsTFA) centrifugation solution [31] and adjusted to an average starting density of ~1.789 g mL^−1^ that corresponded to a refractive index of 1.3724 ± 0.0001, which was determined using an AR200 refractometer (Reichert, Buffalo, NY, USA). The RNA-loaded centrifugation solution was filled into 6 mL crimp top ultracentrifugation tubes (Sorvall, Waltham, MA, USA) and centrifuged at ~130,000× *g* and 20 °C for 65 h using a 65 V13 vertical rotor (Sorvall, Waltham, MA, USA) [35]. After centrifugation, density gradients were fractionated into 15 fractions (each ~400 μL) as described previously [31]. For each fraction, the density was determined by measuring the refraction of the solution, which was correlated against a previously established calibration curve. The last two fractions, usually containing water, were excluded from the analysis. RNA from each fraction was recovered as described previously [31] and stored at −80 °C until further analysis. This density-dependent RNA fractionation was repeated three times independently for each RNA pool, i.e., in three technical replicates.

Total RNA obtained from each fraction was finally converted to cDNA as described above.

### 2.4. Quantification of 16S rRNA in Gradient Fractions

Quantity of bacterial 16S rRNA in each density fraction was measured by quantitative polymerase chain reaction (RT-qPCR) [36] in a two-step assay using reverse transcribed RNA (cDNA) as template. Amplification reactions were performed in qPCR triplicates according to a previously published method [31]. Afterwards, RNA concentrations were calculated by means of correlation to an internal standard curve generated from serial dilutions of a calibrator pool consisting of equal amounts of genomic DNA isolated from *Faecalibacterium prausnitzii* A2-165, *Escherichia coli* DH5-α, *Lactobacillus plantarum* WCSF1 and *Clostridium perfringens* CH05 using the Rotor-Gene Software (version 1.6; Qiagen, Valencia, CA, USA). RNA concentrations in each gradient fraction were averaged across the qPCR triplicates and expressed as percentage RNA concentration relative to the maximum quantity detected among all fractions within the gradient [32].

### 2.5. 16S rRNA Amplicon Library Construction and Sequencing

To assess the general caecal microbiota structure in each individual mouse prior to density-dependent separation, 16S rRNA gene amplicon libraries were constructed from the respective reverse transcribed caecal RNA samples.

For the identification of distinct bacterial taxa involved in [U^13^C] starch assimilation, 16S rRNA gene amplicon libraries were constructed from density-separated and reverse-transcribed RNA obtained from selected SIP fractions. SIP fractions 8–10 contained the bulk of 16S rRNA present in samples from control mice that did not receive any [U^13^C] starch (0 h). These fractions were considered to represent unlabeled bacterial populations and were thus defined ‘light’ fractions. In caecal samples of mice fed with [U^13^C] starch, gradient fractions 5–7 contained the bulk of RNA, which was consequently considered to be ‘heavy’, i.e., ^13^C-labeled, representing bacteria that particularly assimilated the [U^13^C] starch and sequestered the isotope label in their RNA.

All amplicon libraries were prepared by NZGL Genomics Limited (Palmerston North, New Zealand) and comprised a segment of the V3 and V4 regions of the bacterial 16S rRNA gene, which was amplified in a dual-indexed single step PCR reaction and resulted in amplicons of ~459 bp. Unique indexed libraries were quality checked with a Bioanalyzer DNA 1000 LabChip (Agilent Technologies, Inc., Santa Clara, CA, USA), quantified by fluorometric measurements using a Qubit dsDNA HS assay kit (Life Technologies, Camarillo, CA, USA) and finally pooled in equimolar amounts for sequencing on a Illumina MiSeq platform using Illumina MiSeq 500 cycle Kit_V2 chemistry (2 × 250 base PE; Illumina, San Diego, CA, USA).

### 2.6. 16S Ribosomal RNA Phylogenetic Analysis and Statistics

Amplicon sequence reads were processed using QIIME 1.8 (available on: http://qiime.sourceforge.net/) [37] as previously described [32]. 16S rRNA obtained from the individual mouse caecal contents were sequenced in duplicate technical replicates, while 16S rRNA obtained from each of the three ‘light’ (5–7) and three ‘heavy’ (8–10) gradient fractions were sequenced in triplicate, respectively. However, variation between technical replicates were minimal, so sequence reads from technical replicates were analyzed together in order to maximize the sequencing depth.

Alpha diversity in gradient fractions was calculated using Faith’s phylogenetic diversity estimate across 10 iterations based on the minimum number of reads (5646) implemented through the core_diversity_analyses.py script.

Statistical tests of the sequencing data were conducted in R 3.3.2 [38]. A taxon was considered to be ^13^C-labeled when its proportion in ‘heavy’ gradient fractions was significantly higher than in the ‘light’ counterpart fractions at each time point. Therefore, differences in the taxon abundance between ‘heavy’ and ‘light’ were analysed by non-parametric permutation ANOVA using density as factor. The tests were implemented using the perm.anova function in the RVAideMemoire package for R [39] with 2000 permutations. Differences in alpha diversity were analysed using two-factor ANOVA with time and density as factors. All detected differences with a *p*-value ≤ 0.05 were considered significant, while trends with limited significance were defined as *p* > 0.05 but ≤ 0.10. Hierarchical cluster analysis of bacterial profiles retrieved from ‘heavy’ and ‘light’ fractions was performed using distances calculated from centered Pearson’s correlation and average linkage clustering.

In addition to the classification results obtained by QIIME, the three most abundant OTUs of bacterial taxa that differed significantly (*p* ≤ 0.05) between ‘light’ and ‘heavy’ RNA fraction, were classified using BLAST [40]. The relatedness of the respective OTUs to the next cultured bacterial species suggested by BLAST was visualized in a dendrogram created with the CLC Genomic Workbench (version 8.5.2; Qiagen, Hilden, Germany) using the neighbor joining algorithm, Kimura80 as nucleotide distance measure and 10.000 replications for the bootstrap analysis.

All sequencing data obtained in this study were submitted to GenBank and are publicly available under the accession number PRJNA412932.

### 2.7. Organic Acid Analysis and Isotope Labeling Detection

Organic acid concentrations were analysed by gas chromatography (GC). Caecal RNAlater supernatants were diluted five-fold with 0.01 M PBS containing 2-ethylbutyric acid (6.25 mM) as an internal standard. A 500 µL aliquot of the diluted supernatant was acidified with 250 µL concentrated hydrochloric acid and 1000 µL diethyl ether were added. After thorough mixing to allow acids to solubilise in the diethyl ether, the sample was centrifuged at 10,000× *g* for 5 min at 4 °C to complete phase separation. The diethyl ether phase was stored at −80 °C until derivatisation for GC-analysis with flame ionization detection (FID) (for organic acids concentrations) and GC-mass spectrometry (MS) (for isotope analysis).

Organic acid concentrations were quantified by GC-FID based on a previously published method [41]. In a capped GC vial, 100 µL of the diethyl ether phase was derivatised with 20 µL *N*-*tert*-butyldimethylsilyl-*N*-methyltrifluoroacetamide (MSTFA) with 1% *tert*-butyldimethylchlorosilane (Sigma-Aldrich, Auckland, New Zealand) by heating to 80 °C in a water bath for 20 min. To allow complete derivatisation, the samples were left for 48 h at room temperature before analysis. Standards containing 2-ethylbutyric acid (5 mM) as an internal standard were prepared for derivatisation alongside the samples. Analysis was performed on a capillary GC system (GC-2010 Plus; Shimadzu, Kyoto, Japan) equipped with a FID and fitted with a Restek column (Rtx-1, 30 m × 0.25 mm × 0.25 µm) (Bellefonte, PA, USA). The carrier gas was helium with a total flow rate of 21.2 mL/min and pressure of 131.2 kPa. Make-up gas was nitrogen. The temperature program began at 70 °C increasing to 115 °C at 6 °C/min, with a final increase to 300 °C at 60 °C/min, holding for 3 min. Flow control mode was set to linear velocity of 37.5 cm/s. The injector temperature was 260 °C and detector temperature was 310 °C. Samples were injected (1 µL) with a split injection (split ratio: 10:1). The GC instrument was controlled and data processed using Shimadzu GC Work Station LabSolutions Version 5.3 software. Organic acids were quantified by comparison with standard curves generated from authentic standards run in the same batch.

Isotope analysis was performed using diethyl ether extracts as described above, evaporated to dryness, derivatised with 50 μL of methoxyamine, 20 mg/mL) at 40 °C for 90 min, then with MSTFA (80 μL) at 40 °C for 30 min and analysed on an Agilent 7890B GC system coupled to an Agilent 5977A MSD mass spectrometer (Agilent Technologies, Inc., Santa Clara, CA, USA) fitted with a Restek column (Rxi-5ms, 30 m × 0.25 mm × 0.25 µm) (Bellefonte, PA, USA). The carrier gas was helium at 8.8 psi with an average linear velocity of 39.6 cm/s. Samples were injected (1 µL) with a split injection (split ratio: 10:1). The injection temperature was 250 °C, with oven temperature of 35 °C for 2 min, then increasing to 90 °C at 5 °C/min, and to 300 °C at 15 °C/min. Electron ionisation energy was 70 eV. Authentic standards were run alongside, as described for GC-FID above. Compounds were identified using the retention times of authentic standards run in the same batch, and masses by comparison with the NIST11 mass spectral database. Mass spectra were used, in the absence of established Vienna Pee Dee Belemnite ^13^C mass standard, to calculate atom percent excess (APE) according to Equation (1) [42] where (atom %)_E_ is the ^13^C abundance of the RS-fed samples at times 2 h and 4 h, and (atom %)_B_ is the ^13^C abundance of the baseline (time 0 h) samples. The (atom %) values were calculated according to Equation (2) [42], from the following *t*-butyl-dimethyl-silyl organic acid derivative *m*/*z* ratios: acetate, M-119/117; butyrate, M-147/145; and lactate, M-119/117. We were unable to resolve propionate from a contaminating peak with this method.

(1) APE = (atom %)_E_ − (atom %)_B_

(2) Atom % ^13^C = ([^13^C]/([^12^C] + [^13^C])) × 100

## 3. Results

### 3.1. General Microbiota Structure in the Used Animals Prior to Gradient Separation

To determine the general bacterial microbiota composition prior to the density-dependent separation of extracted RNA, 16S rRNA sequences of individual caecal contents of all mice used in the experiment were analyzed.

Sequencing analysis of these samples yielded a total of 75,549 paired-end sequences. The average number of reads per sample were 8394 ± 2148 (range 5646–12,512 reads). Collectively, these sequences were assigned to 69 bacterial genera affiliated with 33 families, 19 orders, 14 classes and 7 phyla, representing the entire caecal community across all samples.

*Firmicutes* and *Bacteroidetes* were the most prevalent phyla within the caecal communities, whereas *Proteobacteria* as well as unclassified *Bacteria* represented minor taxa (Supplementary Table S1). Although a slight variation between individual mice was observed at lower taxonomic levels, *Lachnospiraceae* and *Ruminococcaceae* were the most prevalent families (Supplementary Table S1). The most dominant taxa classified to the most detailed taxonomic level available were unclassified *Lachnospiraceae*, followed by unclassified *Clostridiales*, unclassified *Ruminococcaceae* and *Prevotella* spp. (Supplementary Figure S1).

### 3.2. Density Gradient Formation and Recovery of Stable Isotope-Labeled Bacterial 16S rRNA

To identify intestinal bacteria involved in the assimilation of [U^13^C] starch directly in their natural habitat, RNA isolated from caecal contents and pooled for groups of three mice, each, after 0 h, 2 h and 4 h of feeding with [U^13^C] starch, were density-separated in order to detect ^13^C-labeled bacterial RNA.

**Figure 1:**
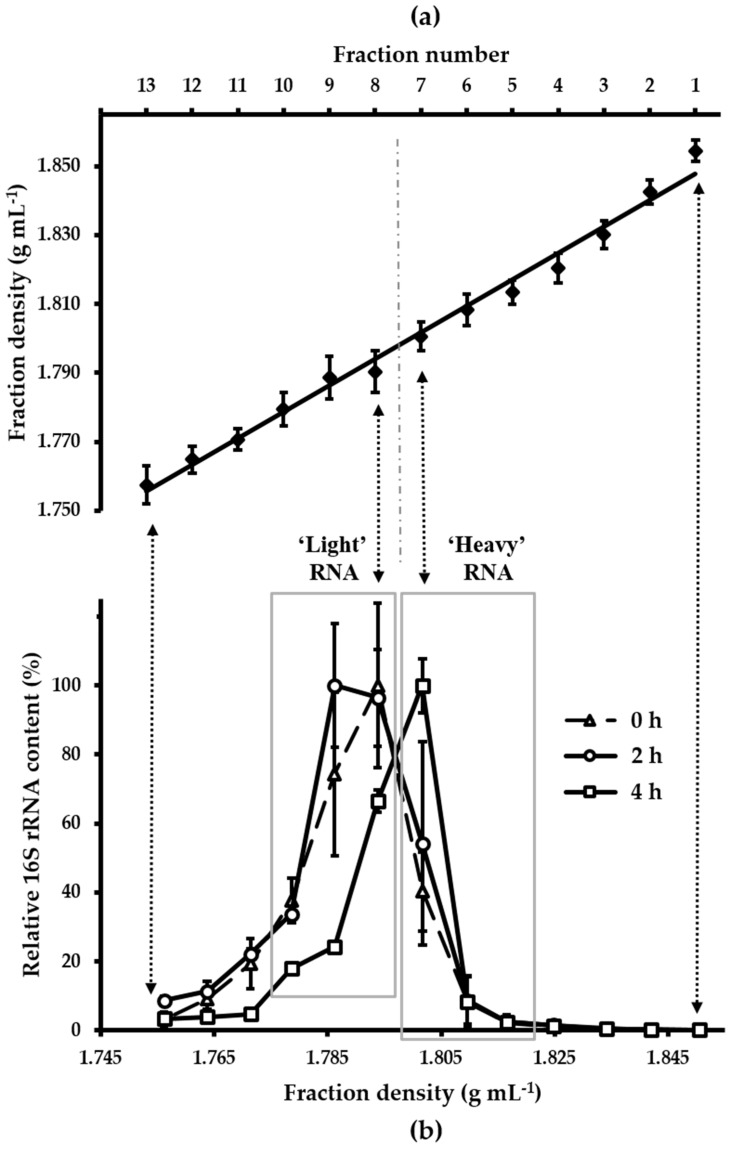
(**a**) Density of isopycnic density gradient fractions. Means and standard errors (SEM) of nine gradients are shown. For each fraction, the SEM was ≤0.006 g mL^−1^. The vertical dash dot line divides the density gradient into ‘heavy’ (≤fraction 7) and ‘light’ (≥fraction 8) fractions; (**b**) Distribution of relative amounts of 16S rRNA. RNA from the isopycnic density gradients was collected before (0 h) or after administration of [U^13^C] starch (2 h and 4 h). Means and SEMs of three technical replicates for each gradient fraction per sampling time are shown. To facilitate comparison between the different gradients, RNA content is calculated as a proportion (%) of the fraction containing the highest RNA concentration (100%) per gradient [32]. Boxes indicate ‘heavy’ and ‘light’ density fractions analyzed for microbiota composition. Vertical arrows indicate corresponding fraction numbers in (**a**,**b**).

The fractionated gradients showed a linearly decreasing density between 1.757 g mL^−1^ (fraction 13) and 1.854 g mL^−1^ (fraction 1; Figure 1a). Distribution patterns of caecal 16S rRNA sequences within the fractionated density gradients showed that the bulk of RNA isolated from mice that had not received [U^13^C] starch (0 h, control RNA) accumulated in the low density fractions 8–10 (~1.794–1.779 g mL^−1^), with peak amounts being present in fractions 8 and 9 (Figure 1b). In contrast, caecal RNA isolated from mice fed with [U^13^C] starch shifted towards the higher density fractions 5–7 (~1.817–1.802 g mL^−1^) with the maximum amount of RNA detected in fraction 7 after 4 h. Furthermore, although after 2 h the majority of RNA still accumulated in fraction 8 and 9 (~1.794 and ~1.786 g mL^−1^), fraction 7 (~1.802 g mL^−1^) contained more RNA than control fraction 7 (Figure 1b).

### 3.3. Characterisation of Metabolically Active Populations and Identification of Potential [U^13^C] Starch Degraders by Density Gradient Formation and Recovery of Stable Isotope-Labeled Bacterial 16S rRNA

Sequencing analysis of selected ‘light’ and ‘heavy’ gradient fractions yielded a total of 944,665 paired-end sequences. The average number of reads for each sample were 52,481 ± 12,338 (range 34,640–75,739 reads).

Phylogenetic analysis of these samples revealed a complex microbiota structure with *Lachnospiraceae*, unclassified *Clostridiales* and *Ruminococcaceae* 16S rRNA gene sequences relatively dominating the ‘light’ as well as the ‘heavy’ communities (Supplementary Figure S2).

**Figure 2:**
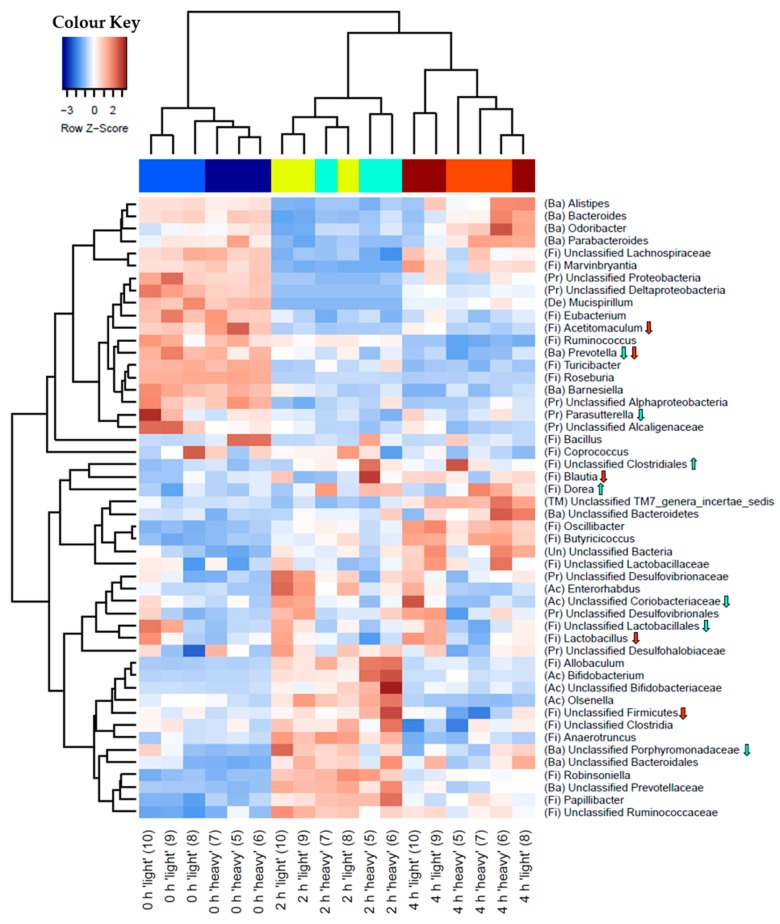
Heatmap of hierarchical clustering of bacterial microbiota composition profiles represented by 16S ribosomal RNA (rRNA) amplicons per sample of ‘heavy’ and ‘light’ gradient fractions at each sampling time. RNA from caecal contents was isolated before (0 h) or after provision of [U^13^C] starch (2 h and 4 h). The presented community profiles are results of three technical replicates for each fraction per sampling time. Bacteria shown represent the 50 taxa with the highest mean relative abundance across all fraction samples. Heatmap colour (blue to dark red) displays the row scaled relative abundance of each taxon across all samples. The number in parentheses indicates the corresponding fraction number. Letters in parentheses preceding taxonomic labels indicate the phylum (Ac = *Actinobacteria*, Ba = *Bacteroidetes*, De = *Deferribacteres*, Fi = *Firmicutes*, Pr = *Proteobacteria*, Ve = *Verrucomicrobia*, Un = Unclassified). Arrows indicate either increase or decrease at *p* ≤ 0.05 in ‘heavy’ fractions compared to the corresponding ‘light’ fractions as calculated by permutation ANOVA with 2000 permutations using density as factor at 2 h (green arrows) and 4 h (red arrows).

Hierarchical cluster analysis revealed community structure profiles of mice 2 h and 4 h after administration of [U^13^C] starch being clearly separated from those detected before the administration of RS (0 h) (Figure 2). In addition, significant differences in Faith´s phylogenetic diversity estimate between ‘heavy’ and ‘light’ fraction samples (*p* = 0.038) as well as between the different sampling times (*p* = 0.023) were detected (Table 1).

**Figure 3:**
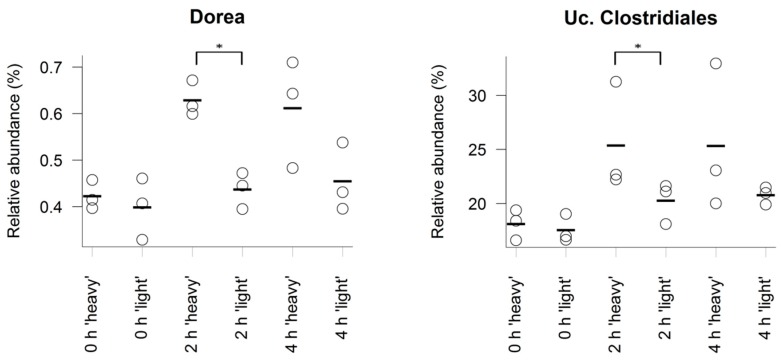
Relative abundance of selected bacterial taxa represented by 16S rRNA amplicons in ‘heavy’ and ‘light’ SIP fractions. RNA from caecal content was isolated before (0 h) or after administration (2 h) of the [U^13^C] starch. Taxa shown display a significantly (*p* ≤ 0.05) higher mean relative abundance in the ‘heavy’ gradient fractions compared to the corresponding ‘light’ fraction samples at 2 h. Points indicate relative abundance in (%) of the total community (*n* = 3 fractions, each), and lines indicate the mean. * Indicates permutation ANOVA significance with 2000 permutations in relative proportions at *p* ≤ 0.05 using density as factor.

To identify bacteria possibly involved in the degradation of the [U^13^C] starch, sequencing profiles retrieved form ‘heavy’ and ‘light’ SIP fractions at each sampling time were screened for relative differences of individual taxa; 2 h after administration of [U^13^C] starch, taxa that showed significantly increased proportions in ‘heavy’ fractions compared to the corresponding ‘light’ counterparts included unclassified *Clostridiales* (*p* = 0.03) and *Dorea* spp. (*p* = 0.03). Taxa that tended towards significant increases included *Bacteroidaceae* (*p* = 0.07), *Bacteroides* spp. (*p* = 0.06) and *Turicibacter* spp. (*p* = 0.08). All these groups also showed higher abundances in the ‘heavy’ RNA 4 h after starch administration, however, not with statistical significance (Figure 2 and Figure 3 and Supplementary Table S2).

At 4 h, the only taxon that was significantly enriched in ‘heavy’ compared to ‘light’ fractions upon RS consumption was *Papillibacter* (*p* = 0.05). However, since this genus also tended to be enriched in heavy RNA before starch, i.e., label addition, we did not consider this genus as a potential starch degrader here (Supplementary Table S2).

**Figure 4:**
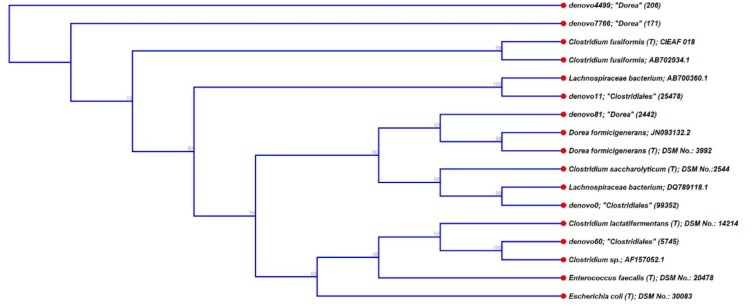
Dendrogram showing the relatedness of the three most abundant OTUs of the bacterial taxa that differed significantly (*p* ≤ 0.05) between ‘light’ and ‘heavy’ RNA fractions (i.e., unclassified *Clostridiales* and *Dorea* spp.) to their next cultured relatives according to a BLAST search. *Escherichia coli* and *Enterococcus faecalis* were used as outgroups.

A BLAST-based phylogenetic analysis (Figure 4) of the three most abundant OTUs of the bacterial taxa that differed significantly (*p* ≤ 0.05) between ‘light’ and ‘heavy’ RNA fractions, indicated affiliations with *Clostridium lactatifermentans* and the family *Lachnospiraceae* (in case of OTUs designated as ‘unclassified *Clostridiales*’) and with *Clostridium fusiformis* and *Dorea formicigenerans* (in case of OTUs designated as *Dorea* spp.).

Finally, proportions of a diverse array of sequences affiliated with unclassified *Porphyromonadaceae*, *Prevotella*, and *Lactobacillus* (all *p* ≤ 0.05), as well as unclassified *Ruminococcaceae*, unclassified *Desulfovibrionaceae* (all *p* ≤ 0.1) and other taxa with smaller population size (≤1% relative abundance) showed reduced relative abundances in the ‘heavy’ fractions compared to the ‘light’ fractions at 2 h and at 4 h (Figure 2 and Supplementary Table S2).

### 3.4. Organic Acid Production and Heavy Isotope Incorporation into Metabolic Products

To gain further insight into the metabolic activity of the caecal microbiota, GC-FID and GC-MS analysis was performed in supernatants of caecal contents to monitor ^13^C-labeled metabolite formation during the course of [U^13^C] starch fermentations in each animal. On average, acetate (~11.8 μmol/mL), butyrate (~3.2 μmol/mL), propionate (1.7 μmol/mL), lactate (0.6 μmol/mL), succinate (0.4 µmol/mL) and valerate (0.2 μmol/mL) were the main metabolic end products detected after administration of the [U^13^C] starch dose (average of 2 h and 4 h; Table 2). However, the metabolite concentrations showed inter-animal differences with on average decreasing amounts of metabolites during the course of fermentation (Table 2). Nevertheless, GC-MS analysis revealed label incorporation into acetate, butyrate and lactate (Table 3). Although the amount of ^13^C in the analyzed metabolites, expressed as atom percent excess (APE), showed inter-animal variations, in each mouse, butyrate was the predominant labeled fermentation product, followed by lactate and acetate 2 h after the administration of starch. During the course of fermentation, isotope-labeled butyrate as well as acetate were consistently produced from the [U^13^C] starch, while amounts of ^13^C-labeled lactate concentrations declined after 2 h.

## 4. Discussion

In the last decades, RS has been increasingly investigated for its potential to function as a prebiotic agent with the ability to modulate composition and fermentation profiles of intestinal communities, thereby improving human health and well-being [9,15]. Previous reports, in which RS was administrated to mice, rats or pigs showed alterations of the community by increased abundances of *Bifidobacterium*, *Akkermansia*, *Allobaculum*, *Lactobacillus*, *Ruminococcus*, *Prevotella* and several other groups [17,18,20,43], and also increased amounts of SCFA such as acetate, propionate and butyrate [18,44,45]. In the case of humans, in particular *Ruminococcus bromii* was suggested to play a major role in resistant starch digestion and subsequent butyrate production [46], also based on an RNA-SIP study conducted with material from a bioreactor simulating the human intestinal tract [29]. However, conventional feeding studies might have been biased by enrichments and generally lacked the ability to directly link the conversion of RS to distinct groups of gut bacteria in their natural habitat. Hence, knowledge about the key players directly involved in the break-down of this complex carbohydrate in situ is still scarce.

Here, we report the use of [U^13^C]-labeled potato starch, a source of RS, as a substrate for the caecal community using mice as model system, and the use of RNA-SIP in combination with high-throughput 16S rRNA sequencing and GC-MS analysis to gain phylogenetic information about the bacteria involved in [U^13^C] starch degradation in situ. In a previous in vitro study, we had used RNA-SIP to identify bacterial groups from faecal samples of mice that were involved in the in vitro assimilation of [U^13^C] starch [33]. To the best of our knowledge, the present study represents just the second report on the use of RNA-SIP under in vivo conditions in a vertebrate intestinal system [30], and the first using RS. 

The density range of the obtained isopycnic gradients agreed with gradients from previous RNA-SIP studies and included densities necessary to separate isotope-labeled [^13^C]RNA from unlabeled background RNA [31,32,33]. The observed shifts of 16S rRNA species isolated from [U^13^C] starch-fed mice towards higher densities, 2 h and 4 h after provision of the labeled starch clearly indicated [U^13^C] starch utilization and incorporation of ^13^C into bacterial 16S rRNA. After 2 h of feeding, labeling of RNA was less pronounced, which might be explained with the digestion-resistant nature of RS, the presence of competing [Unlabeled) substrates in the gut, and the fact that in microbial fermentation processes most of the carbon is used in energy metabolism rather than in biosynthesis processes, such as RNA synthesis [28].

The caecal microbiota analysis of all experimental animals based on unseparated RNA revealed an overall bacterial community structure that is in very good alignment with profiles observed in caecal samples of mice analyzed by conventional 16S sequencing [47,48,49], indicating a normal (healthy) microbiota in all animals used for this study. In addition, indicated by a clear delineation in hierarchical clustering analysis before the administration of the RS, as well as during the course of fermentation, it is suggested that RS dynamically introduced changes into this microbiota. This is further supported by a significant increasing diversity estimate, which suggests that more and more species benefited from the available RS and/or its degradation products and/or metabolites over time.

Screening of 16S amplicon sequencing profiles revealed only a limited number of taxa that were significantly increased in abundance in ‘heavy’ vs. ‘light’ gradient fractions, thereby suggesting involvement in the assimilation of the [U^13^C] starch. Interestingly, only a few of them are related to well-known starch degraders.

With limited significance, *Bacteroidaceae* and *Bacteroides* spp. were relatively enriched in ‘heavy’ RNA, isolated 2 h after feeding starch. These taxa comprise a particular high number of members that are associated with the utilization of various types of starch and are, therefore, considered to be one of the major amylolytic species in the human colon [50,51,52]. Starch utilization in *Bacteroides* spp. is mediated through a well-characterized starch binding and degradation system referred to as starch utilization system (SUS) [53]. Hence, our data allow careful speculation that *Bacteroides* spp. might have functioned as initial starch degraders here, producing smaller hydrolysis products, e.g., maltose and glucose that served as substrates for other taxa such as *Dorea* spp. and unclassified *Clostridiales*, which became significantly labeled during the experiment. This is in line with a previous report demonstrating that gut *Bacteroidales* significantly contributed to cross-feeding in intestinal ecosystems [54]. Notably, labeled metabolites for cross-feeding might have also resulted from starch hydrolysis mediated by the host itself in the small intestine through the enzymatic activity of pancreatic α-amylases, mucosal maltase-glucoamylases and sucrose-isomaltases [55]. However, starch fractions from potatoes were shown to be degraded rather slowly in comparison to other botanical sources in vitro and in vivo [56,57].

*Dorea* is one of the most dominant human gut genera [58]. Increases in *Dorea* portions upon a diet enriched with RS were recently reported in mice [59], suggesting that this genus might indeed be stimulated by RS within a complex food web. However, other reports showed a decrease of this genus following RS consumption in humans and pigs [20,60,61]. Notably, *Dorea* species, including *Dorea formicigenerans*, to which the most abundant OTU from this group was affiliated, are not known to be capable of starch hydrolysis so far [62]. Either, the OTUs classified as *Dorea* ssp. here represent so-far uncultured species, being capable of starch hydrolysis, or the observed labeling results from cross-feeding on metabolites released from the labeled starch by other bacteria or by the host.

Both explanations also hold true for the other taxa that were found to be enriched in the ‘heavy’ RNA fraction, as many of them were related to taxa so far not known as starch degraders, such as *Turicibacter* spp. or *Clostridium lactatifermentans* [63,64]. Interestingly, increases in relative abundances of *Turicibacter* spp. were also shown in the hindgut of pigs fed raw potato starch [65] and two of the three most abundant OTUs categorized as ‘unclassified *Clostridiales*’ appear to be affiliated with *Lachnospiraceae*, whose members are known for their capacity to degrade several plant-derived polysaccharides including starch [66].

RS is well-known to significantly increase SCFA production in vitro and in vivo [67,68,69]. In particular, potato starch was associated with elevated butyrate levels in the large bowel of humans, rats and pigs [44,70,71]. Butyrate production can either originate from a direct stimulation of butyrate-producing species, mostly associated with members of the *Clostridium* cluster IV (*Ruminococcaceae* family) and *Clostridium* cluster XIVa (*Lachnospiraceae* family) [72], and/or by cross-feeding between different members of the caecal microbiota [73,74,75]. Similarly, butyrate was the metabolic product showing the highest level of incorporated label in our study. It might have been directly produced from starch by the metabolic activity of bacteria affiliated with unclassified *Clostridiales* and the *Lachnospiraceae* family, which also became significantly labeled during the experiment.

The data obtained here show similarities in comparison to our recently published in vitro data on the fermentation of potato starch by a mice fecal microbiota [33]. While in both experiments, labeled acetate and butyrate were detected, labeled propionate was only detected in vitro, and labeled lactate only in vivo. In vitro, 24 bacterial taxa became significantly ^13^C-labeld, which was now confirmed for *Bacteroides* and unclassified *Clostridiales* also under in vivo conditions, thereby strongly suggesting an actual involvement of these groups in intestinal starch degradation.

## 5. Conclusions

Collectively, our data show that the RNA-SIP approach is generally suited to link the structure and function of intestinal microbiotas also under the in situ conditions of mice intestinal tracts. Heavier, i.e., presumably isotopically labeled RNA, could be extracted only 2–4 h after having fed labeled potato starch to the animals. In addition, a few, but statistically significant differences in microbial community composition between labeled and unlabeled RNA fractions were shown, probably indicating starch-degrading bacteria from the intestinal tract. Bacteria affiliated with *Bacteroides* and *Clostridiales* were confirmed as initial starch degraders in the caecal microbiota of mice, while other bacteria related to *Clostridiales*, *Dorea*, and *Turicibacter* might have played a role as cross-feeders on metabolic products, although a direct involvement in starch degradation cannot be ruled out. Clearly, further studies are needed to verify the role of the bacteria suggested as starch degraders and cross-feeders here in more detail. These studies should account for the limitations of our pilot study, i.e., include, for instance, more test and control animals, more sampling points (to address the effect of cross-feeding in more detail), longer incubation times (to achieve a stronger labeling of RNA and metabolites) and ^12^C-controls (to ensure a nutritional balance between test and control animals). Finally, SIP might be used in a more quantitative way, as recently introduced by Hungate and colleagues [76], albeit for DNA-SIP only.

## Figures and Tables

**Table 1:** Faith’s phylogenetic diversity estimate in ‘heavy’ and ‘light’ density gradient fractions before (0 h) or 2 h and 4 h after administration of [U^13^C] starch.

Faith’s Phylogenetic Diversity Estimate									
	PD Whole Tree								
	0 h		2 h		4 h			*p*-Value	
SIP Fractions	Mean	SEM	Mean	SEM	Mean	SEM	Density	Time	Density × Time
Heavy	43.53	0.42	45.39	1.34	46.35	2.04	0.038 *	0.023 *	0.653
Light	46.86	0.38	45.02	0.47	50.92	1.01			

* Indicates two-factor ANOVA significance at the 5% level with time and density as factors. PD = Phylogenetic diversity.

**Table 2:** Organic acid fermentation products from caecal contents of individual mice before (0 h) and after administration of resistant starch (RS; 2 h and 4 h). M1 to M9 identify the mouse source for caecal contents at each sampling time.

Fermentation Products									
Concentration (μmol/mL Supernatant)									
	0 h			2 h			4 h		
Organic acid (as conjugate base)	M1	M2	M3	M4	M5	M6	M7	M8	M9
Formate (C1)	0.3 ^a^	0.3 ^a^	0.3 ^a^	0.3 ^a^	0.3 ^a^	0.3 ^a^	0.3 ^a^	0.3 ^a^	0.3 ^a^
Acetate (C2)	17.77	16.6	18.7	18.7	13.39	6.69	10.82	7.27	14.22
Propionate (C3)	4.9	2.58	3.08	2.69	1.71	1.35	1.6	1.33	1.75
Butyrate (C4)	5.65	4.72	4.19	4.25	3.04	1.96	3.74	2.14	4.12
Valerate (C5)	0.23	0.17	0.17	0.26	0.22	0.13	0.16	0.12	0.18
Caproate (C6)	0.1 ^a^	0.1 ^a^	0.1 ^a^	0.1 ^a^	0.1 ^a^	0.1 ^a^	0.1 ^a^	0.1 ^a^	0.1 ^a^
Enanthate (C7)	0.1 ^a^	0.1 ^a^	0.1 ^a^	0.1 ^a^	0.1 ^a^	0.1 ^a^	0.1 ^a^	0.1 ^a^	0.1 ^a^
Lactate (C3; 2-OH)	0.59	0.96	3.66	0.94	0.38	0.8	0.67	0.25	0.48
Succinate (C4; 1,4-dicarboxylate)	0.52	0.45	0.82	0.47	0.32	0.31	0.33	0.3 ^a^	0.3 ^a^
Isobutyrate (C4; 2-methyl-C3)	0.25	0.15 ^a^	0.19	0.22	0.15 ^a^	0.15 ^a^	0.15 ^a^	0.15 ^a^	0.15 ^a^
Isovalerate (C5; 3-methyl-C4)	0.13	0.1 ^a^	0.14	0.13	0.1 ^a^	0.1 ^a^	0.1 ^a^	0.1 ^a^	0.1 ^a^

^a^ At or below detection limit (detection limit for each organic acid shown).

**Table 3:** Organic acid fermentation product ^13^C atom percent excess (APE) relative to natural abundance of ^13^C-labeled acid at time 0 h samples, from caecal contents of individual mice after administration of resistant starch (RS; 2 h and 4 h).

^13^C-Labeled Fermentation Products						
^13^C Atom Percent Excess (APE) Relative to Natural Abundance of ^13^C-Labeled Acid at Time 0 H Samples						
	2 h			4 h		
Organic acid (as conjugate base)	M4	M5	M6	M7	M8	M9
Acetate	2.7	0.1	24.6	6.5	20.1	3.3
Propionate	ND	ND	ND	ND	ND	ND
Butyrate	6.4	0.4	39.7	14.8	38.3	8.0
Lactate	6.1	0.0	38.8	3.8	0.7	0.4

M4 to M9 identify the mouse source for caecal contents at each sampling time. ND = Not determined.

## Supplementary Materials

The following are available online at http://www.mdpi.com/2072-6643/10/2/179/s1, Table S1: Relative abundances (%) of bacterial taxa based on 16S ribosomal RNA (rRNA) amplified from density-unseparated RNA isolated from individual caecal content samples before (0 h) and after administration of resistant starch (RS; 2 h and 4 h), Table S2: Relative abundance (%) of those bacterial taxa with significant differences between ‘heavy’ and ‘light’ RNA-SIP fractions extracted from caecal content 2 h and 4 h after administration of [U^13^C]starch, Figure S1: Bacterial microbiota composition based on the relative abundances of 16S ribosomal RNA (rRNA) sequences of genus-level taxa. Density-unseparated RNA was obtained from caecal contents of mice before (0 h) or after administration of [U^13^C]starch (2 h and 4 h), Figure S2: Bacterial microbiota composition represented by the relative abundances of 16S ribosomal RNA (rRNA) sequences of genus-level taxa in ‘heavy’ and ‘light’ density fractions.
