# Supplementary material for: In Vivo Assessment of Resistant Starch Degradation by the Caecal Microbiota of Mice Using RNA-Based Stable Isotope Probing—A Proof-of-Principle Study
Source: Nutrients. 2018 Feb 6;10(2):179. doi: 10.3390/nu10020179 (PMC5852755; doi:10.3390/nu10020179)
Supplement: Supplementary file 1 [file nutrients-10-00179-s001.pdf]

Supplementary Materials

Table S1. Relative abundances (%) of bacterial taxa based on 16S ribosomal RNA (rRNA) amplified from density-unseparated RNA isolated from individual caecal content samples before (0 h) and after administration of resistant starch (RS; 2 h and 4 h).

General microbiota structure									
	Relative abundance (%)								
	0 h			2 h			4 h		
Bacterial taxon (Phylum, Class, Family)	M1	M2	M3	M4	M5	M6	M7	M8	M9
**Firmicutes**	74.60	76.07	76.68	87.52	82.41	77.81	83.25	80.30	81.88
Clostridia	66.81	68.50	68.18	83.62	76.77	72.39	81.31	74.63	78.39
Lachnospiraceae	33.22	38.62	34.32	29.84	26.30	28.69	48.17	27.66	32.66
Ruminococcaceae	20.37	12.97	14.86	23.24	18.91	24.96	17.93	20.78	23.13
Uc. Clostridiales	12.77	16.43	18.35	30.12	30.84	17.92	14.61	25.68	22.11
Erysipelotrichi	5.07	6.05	5.22	2.36	0.94	3.05	0.44	2.43	2.67
Erysipelotrichaceae	5.07	6.05	5.22	2.36	0.94	3.05	0.44	2.43	2.67
Bacilli	2.16	0.65	2.33	1.33	4.08	1.72	1.15	2.64	0.37
Lactobacillaceae	2.03	0.58	2.07	1.29	3.78	1.54	1.05	2.46	0.32
Uc. Lactobacillales	0.13	0.05	0.26	0.03	0.30	0.18	0.10	0.19	0.03
**Bacteroidetes**	18.92	16.67	17.88	8.85	12.94	16.24	12.10	14.65	12.33
Bacteroida	18.85	16.59	17.77	8.82	12.91	16.21	11.92	14.54	12.20
Prevotellaceae	9.19	5.15	7.95	5.24	7.90	8.66	5.19	6.05	3.74
Porphyromonadaceae	6.56	5.63	7.04	3.24	4.35	5.33	3.86	5.66	4.71
Bacteroidaceae	1.83	2.96	1.14	0.04	0.20	0.76	1.54	1.27	1.13
Rikenellaceae	1.06	2.64	1.45	0.24	0.29	1.10	1.13	1.22	2.47
Uc. Bacteroidales	0.21	0.22	0.19	0.06	0.17	0.35	0.20	0.35	0.13
**Proteobacteria**	4.11	3.36	3.12	2.13	2.18	2.69	1.47	2.22	2.67
Deltaproteobacteria	2.30	2.18	2.10	2.07	1.72	2.07	1.05	1.70	2.37

Desulfovibrionaceae	1.98	0.08	1.73	1.77	1.41	1.79	0.14	1.32	2.02
Uc. Desulfovibrionales	0.25	0.03	0.34	0.26	0.29	0.19	0.03	0.32	0.31
Betaproteobacteria	1.56	0.80	1.00	0.06	0.42	0.55	0.34	0.52	0.25
Alcaligenaceae	1.55	0.79	0.98	0.06	0.38	0.55	0.33	0.52	0.24
**Uc. Bacteria**	2.24	2.68	2.15	0.59	1.72	2.64	2.44	2.47	2.55
**Actinobacteria**	0.12	0.17	0.09	0.89	0.73	0.55	0.16	0.29	0.28
Actinobacteria	0.12	0.17	0.09	0.89	0.73	0.55	0.16	0.29	0.28
Coriobacteriaceae	0.11	0.03	0.09	0.13	0.26	0.16	0.16	0.11	0.06
Bifidobacteriaceae	0.01	0.14	-	0.76	0.47	0.39	-	0.19	0.22
**TM7**	0.01	0.03	0.09	0.02	0.03	0.07	0.06	0.07	0.29
**Deferribacteres**	-	1.02	-	-	-	-	0.53	-	-

M1 to M9 identify the mouse source at each sampling time. Uc. = Unclassified; - = not detected.

Table S2. Relative abundance (%) of those bacterial taxa with significant differences between 'heavy' and 'light' RNA-SIP fractions extracted from caecal content 2 h and 4 h after administration of [U^{13}C]starch. For comparison, relative abundances before starch administration (0 h) is also shown.

	**0 h**					**2 h**					**4 h**				
	Heavy		Light			Heavy		Light			Heavy		Light		
Bacterial taxon	Mean	SEM	Mean	SEM	*p*-value	Mean	SEM	Mean	SEM	*p*-value	Mean	SEM	Mean	SEM	*p*-value
**Firmicutes**	78.82	0.32	75.45	1.27	0.03 *	80.41	1.7	77.73	0.86	0.25	81.66	1.75	80.05	1.48	0.49
Uc. Firmicutes	0.14	0	0.16	0.01	0.13	0.21	0.03	0.17	0	0.40	0.1	0.01	0.16	0.01	0.05 *
Uc. Lactobacillales	0.03	0	0.06	0.01	0.27	0.03	0	0.05	0.01	0.04 *	0.02	0	0.05	0.01	0.06 (*)
Lactobacillaceae	0.71	0.05	0.88	0.15	0.31	0.66	0.09	0.88	0.13	0.25	0.71	0.08	1.04	0.09	0.07 (*)
Lactobacillus	0.69	0.06	0.86	0.15	0.35	0.64	0.09	0.85	0.12	0.25	0.67	0.07	1.00	0.08	0.04*
Uc. Clostridiales	18.12	0.81	17.54	0.75	0.78	25.39	2.94	20.27	1.1	0.03 *	25.35	3.91	20.78	0.47	0.28
Incertae Sedis XIV	0.01	0.00	0.01	0.00	0.59	0.01	0.01	0.01	0.00	0.60	0.01	0.00	0.01	0.00	0.03 *
Blautia	0.01	0	0.01	0	0.58	0.01	0.01	0.01	0	0.58	0.01	0	0.01	0	0.02 *
Lachnospiraceae	36.52	0.67	36.09	1.14	0.69	25.52	0.98	26.77	0.67	0.42	31.38	2.11	33.10	1.03	0.41
Acetitomaculum	0.08	0.01	0.05	0	0.15	0.01	0	0.01	0	0.21	0.01	0	0.03	0.01	0.02 *
Dorea	0.42	0.02	0.4	0.04	0.69	0.63	0.02	0.44	0.02	0.03 *	0.61	0.07	0.45	0.04	0.18
Ruminococcaceae	17.24	1.33	14.87	0.55	0.09 (*)	22.52	1.23	25.60	0.74	0.06 (*)	20.94	1.22	22.00	1.00	0.53
Uc. *Ruminococcaceae*	11.59	1.20	9.38	0.26	0.06 (*)	16.14	0.89	18.91	0.86	0.06 (*)	14.69	0.82	15.46	0.70	0.54
Papillibacter	0.03	0	0.02	0	0.09 (*)	0.08	0.01	0.06	0	0.16	0.06	0	0.04	0	0.05 *
Erysipelotrichaceae	5.51	0.17	5.41	0.07	0.73	5.57	0.42	3.52	0.05	0.07 (*)	2.74	0.33	2.54	0.16	0.69
Turicibacter	5.36	0.17	5.33	0.07	1.00	3.73	0.35	2.57	0.07	0.08 (*)	2.37	0.24	2.22	0.09	0.59
**Bacteroidetes**	17.12	0.27	19.07	0.71	0.02 *	15.47	1.23	17.19	0.61	0.33	14.1	1.24	14.63	1.61	0.81
Bacteroidaceae	0.86	0.07	0.88	0.03	0.84	0.41	0.05	0.24	0.04	0.07 (*)	0.92	0.19	0.66	0.23	0.44
Bacteroides	0.86	0.07	0.88	0.03	0.85	0.41	0.05	0.24	0.04	0.06 (*)	0.92	0.19	0.66	0.23	0.49
Porphyromonadaceae	10.30	0.20	11.25	0.55	0.16	9.46	0.45	10.44	0.61	0.32	9.34	0.54	8.98	0.83	0.78
Uc. *Porphyromonadaceae*	3.94	0.03	4.66	0.39	0.11	4.93	0.28	5.81	0.37	0.04 *	4.5	0.31	4.67	0.45	0.70
Prevotellaceae	4.86	0.44	5.75	0.40	0.21	4.90	0.69	5.87	0.24	0.23	2.60	0.39	3.73	0.27	0.10 (*)
Prevotella	4.12	0.39	4.84	0.4	0.33	2.15	0.29	2.98	0.15	0.05 *	1.34	0.19	2.07	0.17	0.01 *

**Proteobacteria**	2.84	0.11	3.87	0.49	0.12	2.29	0.3	3.16	0.26	0.16	2.3	0.24	3.01	0.19	0.05 *
Alcaligenaceae	0.67	0.04	1.27	0.19	0.08 (*)	0.35	0.04	0.52	0.07	0.14	0.33	0.04	0.59	0.10	0.04 *
Parasutterella	0.03	0.01	0.06	0.02	0.24	0.01	0	0.02	0	0.04 *	0.03	0.01	0.03	0.01	0.55
Uc. *Desulfovibrionales*	0.11	0.00	0.12	0.02	0.84	0.13	0.01	0.15	0.02	0.24	0.12	0.02	0.16	0.02	0.10 (*)
Desulfohalobiaceae	0.02	0.00	0.01	0.00	0.41	0.02	0.00	0.02	0.00	0.15	0.01	0.00	0.02	0.00	0.10 (*)
Desulfovibrionaceae	1.37	0.08	1.61	0.20	0.38	1.71	0.24	2.43	0.18	0.05 *	1.58	0.15	1.93	0.13	0.15
Uc. *Desulfovibrionaceae*	1.37	0.08	1.61	0.20	0.33	1.71	0.24	2.42	0.18	0.06 (*)	1.58	0.15	1.93	0.13	0.16
**Actinobacteria**	0.12	0.01	0.15	0.01	0.14	0.53	0.11	0.45	0.04	0.59	0.12	0.02	0.21	0.05	0.14
Coriobacteriaceae	0.09	0.01	0.13	0.01	0.07 (*)	0.20	0.02	0.31	0.03	0.03 *	0.06	0.00	0.17	0.05	0.00 *
Enterorhabdus	0.04	0.01	0.06	0.01	0.24	0.08	0.01	0.19	0.02	0.09 (*)	0.04	0	0.11	0.03	0.06 (*)
Uc. *Coriobacteriaceae*	0.02	0.01	0.03	0	0.25	0.02	0.01	0.05	0.01	0.03 *	0.01	0	0.04	0.02	0.10 (*)

Values represent mean and SEM based on three 'heavy' and three 'light' gradient fractions per time point, respectively. *p*-values indicate permutation ANOVA significance with 2000 permutations using density as factor. * = $p \leq 0.05$; (*) = $p \leq 0.1$. Uc. = Unclassified. Highlighted groups are mentioned in the text.

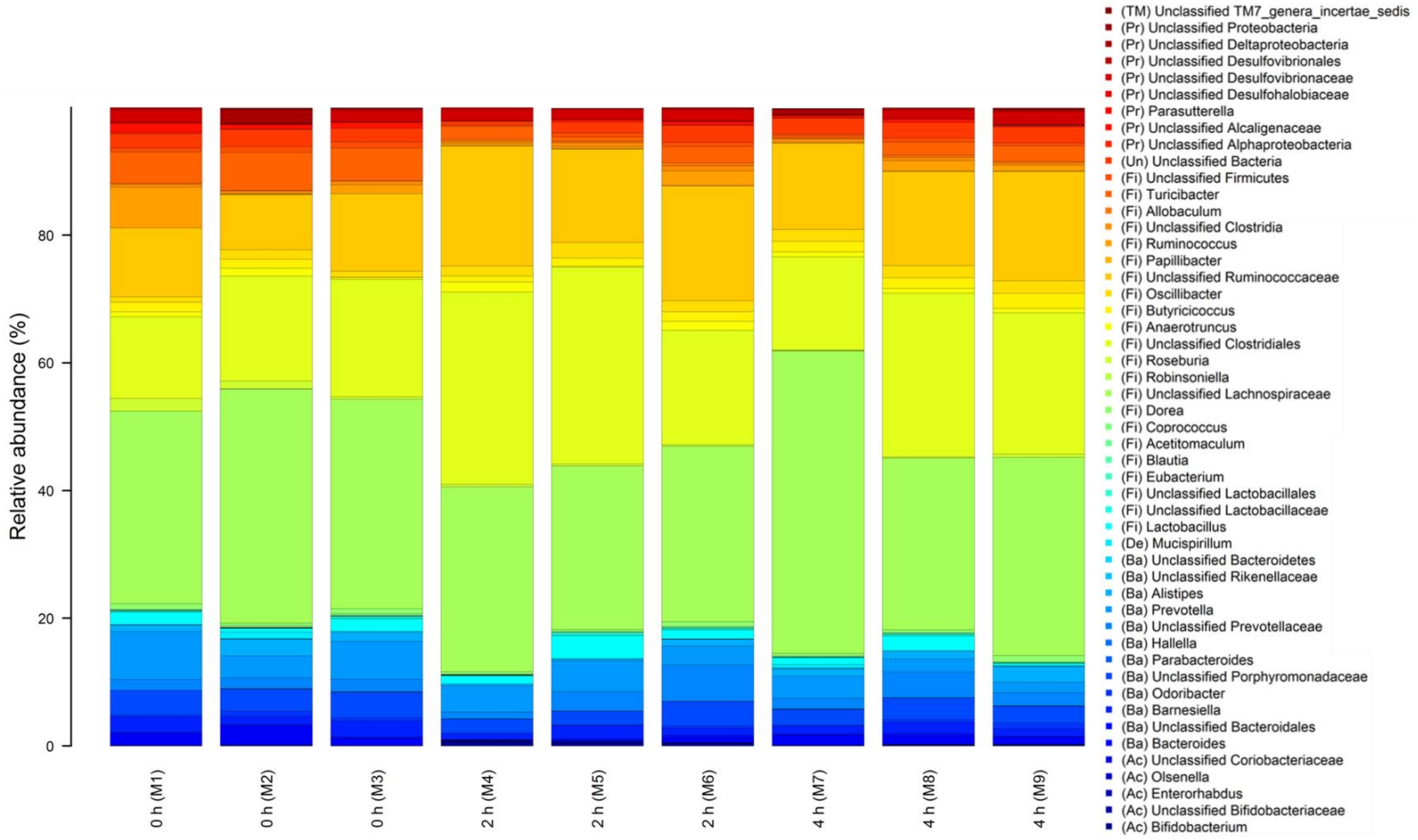

Figure S1. Bacterial microbiota composition based on the relative abundances of 16S ribosomal RNA (rRNA) sequences of genus-level taxa. Density-unseparated RNA was obtained from caecal contents of mice before (0 h) or after administration of [$U^{13}C$] starch (2 h and 4 h). M1 to M9 identify the mouse source for caecal contents. Letters in parentheses preceding taxonomic labels indicate the phylum (Ac = *Actinobacteria*, Ba = *Bacteroidetes*, De = *Deferribacteres*, Fi = *Firmicutes*, Pr = *Proteobacteria*, Ve = *Verrucomicrobia*, Un = Unclassified). Bacteria shown represent the 50 taxa with the highest mean relative abundance across all samples.

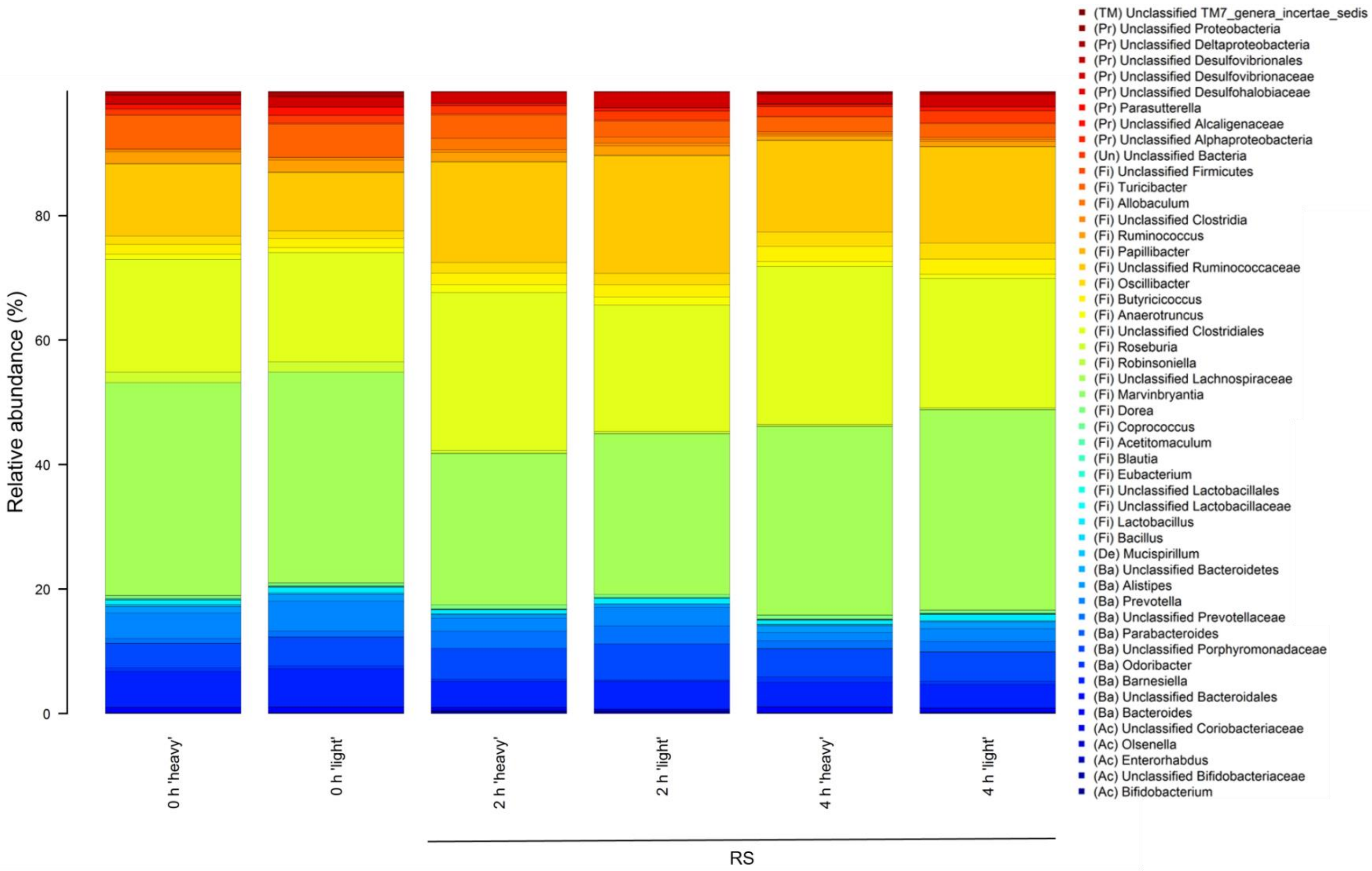

Figure S2. Bacterial microbiota composition represented by the relative abundances of 16S ribosomal RNA (rRNA) sequences of genus-level taxa in 'heavy' and 'light' density fractions. RNA from caecal content was isolated at 0 h (before) or 2 h and 4 h after administration of [U^{13}C] starch. Shown community profiles are based on three technical replicates for each fraction per sampling time and represent the 50 taxa with the highest mean relative abundance across all fraction samples. Letters in parentheses preceding taxonomic labels indicate the phylum (Ac = *Actinobacteria*, Ba = *Bacteroidetes*, De = *Deferribacteres*, Fi = *Firmicutes*, Pr = *Proteobacteria*, Ve = *Verrucomicrobia*, Un = Unclassified).
